# How Do Changes to the Railroad Causeway in Utah’s Great Salt Lake Affect Water and Salt Flow?

**DOI:** 10.1371/journal.pone.0144111

**Published:** 2015-12-07

**Authors:** James S. White, Sarah E. Null, David G. Tarboton

**Affiliations:** 1 Department of Watershed Sciences, Utah State University, Logan, Utah, United States of America; 2 Department of Civil and Environmental Engineering, Utah State University, Logan, Utah, United States of America; University of Vigo, SPAIN

## Abstract

Managing terminal lake elevation and salinity are emerging problems worldwide. We contribute to terminal lake management research by quantitatively assessing water and salt flow for Utah’s Great Salt Lake. In 1959, Union Pacific Railroad constructed a rock-filled causeway across the Great Salt Lake, separating the lake into a north and south arm. Flow between the two arms was limited to two 4.6 meter wide rectangular culverts installed during construction, an 88 meter opening (referred to locally as a breach) installed in 1984, and the semi porous material of the causeway. A salinity gradient developed between the two arms of the lake over time because the south arm receives approximately 95% of the incoming streamflow entering Great Salt Lake. The north arm is often at, or near, salinity saturation, averaging 317 g/L since 1966, while the south is considerably less saline, averaging 142 g/L since 1966. Ecological and industrial uses of the lake are dependent on long-term salinity remaining within physiological and economic thresholds, although optimal salinity varies for the ecosystem and between diverse stakeholders. In 2013, Union Pacific Railroad closed causeway culverts amid structural safety concerns and proposed to replace them with a bridge, offering four different bridge designs. As of summer 2015, no bridge design has been decided upon. We investigated the effect that each of the proposed bridge designs would have on north and south arm Great Salt Lake elevation and salinity by updating and applying US Geological Survey’s Great Salt Lake Fortran Model. Overall, we found that salinity is sensitive to bridge size and depth, with larger designs increasing salinity in the south arm and decreasing salinity in the north arm. This research illustrates that flow modifications within terminal lakes cannot be separated from lake salinity, ecology, management, and economic uses.

## Introduction

Managing lake elevation and salinity are growing problems for terminal lakes worldwide [1]. Many terminal lakes have become smaller and more saline in recent decades, often as water diversions have reduced streamflow contributions. For example, this has occurred in Iran’s Lake Urmia, California’s Mono Lake, Nevada’s Walker Lake, and Utah’s Great Salt Lake (GSL). Lake Urmia and GSL also have solid-fill causeways that limit salt and water exchange throughout the lakes. Better understanding how potential changes to terminal lake causeways may alter water and salt flow may improve causeway designs, maintain terminal lake ecology and commercial uses, and provide opportunities to manage salinity in terminal lakes. We focus on GSL as our study lake.

GSL is a pluvial lake and a remnant of the larger, historical Lake Bonneville. It is the largest saline lake in the western hemisphere and the fourth largest in the world [2]. GSL’s large population of macroinvertebrates supports millions of resident and migratory birds, making the lake a vital link in the Pacific Flyway [3]. The lake also contributes approximately $1.3 billion annually to Utah’s economy through recreation, mineral extraction, and brine shrimp harvest [4]. Because of its ecological, social, and economic significance, lake elevation and salinity are important to local residents, tourists, lake managers, and stakeholders.

In 1959, Union Pacific Railroad constructed a rock-filled, semi-porous railroad causeway across GSL, bisecting the lake into north and south bays, locally referred to as “arms”. Since that time, lake dynamics have changed dramatically, with substantial salinity differences between the two arms. Salinity in the north arm is often saturated (averaging approximately 317 g/L), while the south arm, which receives nearly all streamflow, averages less than half the salinity of the north [5]. Two 4.6 meter (m) rectangular culverts were originally built to maintain boater recreation, but also allowed bi-directional flow through the causeway (south to north flow is from the elevation gradient and north to south flow is from the density gradient). The culverts subsided into soft lakebed sediments and were filled in 2012 and 2013. A bridge in the causeway has been proposed as a replacement, and four trapezoidal bridge designs were provided by Union Pacific Railroad [6]. The design of the bridge will likely change water and salt flow, with potential to significantly alter salinity levels in each arm.

We investigated the salt and water balance between GSL’s north and south arms from anticipated railroad causeway alterations by updating and applying US Geological Survey’s (USGS) Great Salt Lake Fortran Model [7–9]. We model elevation, salinity, and total salt of GSL’s north and south arms with historical and current causeway conditions, the four bridge alternatives, and a “whole lake” alternative without a causeway. Our research evaluates causeway alternatives to identify promising solutions for managing water and salt flow in GSL. While specific salinity targets have not been identified for GSL, salinities that support brine shrimp are a priority to maintain ecosystems and industry [10]. This study also provides a modeling approach to aid decision-making for other terminal lakes divided by causeways.

Computer models provide tools to evaluate and predict changes to hydrologic and environmental systems. The relative simplicity of many closed basin systems enables a mass balance approach to simulating hydrologic conditions. Mass balance models have been used to investigate lake dynamics at other terminal lakes, including Mono Lake [11], Argentina’s Laguna Mar Chiquita [12], Ethiopia’s Lake Tana [13] and Kazakhstan’s Aral Sea [14]. These models help inform management decisions, such as inflow quantities necessary to maintain desired salinity levels.

USGS’ GSL Fortran Model was developed to evaluate GSL water balance and salinity conditions. It has been updated several times since its creation [7], primarily to account for changes in causeway condition. For example, after the causeway was constructed, frequent additions of fill material to prevent causeway flooding reduced water conveyance through the causeway. Also, an 88 m long breach was installed in 1984. The GSL Fortran model was revised and recalibrated in 1997 following these changes [8]. The most recent update added the West Desert Pumping Project [9], constructed in 1986 to alleviate flooding of nearby infrastructure by pumping lake water into the desert. We updated the GSL Fortran Model to accommodate new trapezoidal causeway bridge alternatives and evaluate how proposed causeway changes affect the water and salt balance in GSL. This research quantifies lake salinity and elevation with proposed causeway modifications, information that can directly aid decision-making and management of GSL.

In the following sections of this paper, we describe GSL geography, hydrology, anthropogenic impacts, and ecology. Next we explain the GSL Fortran Model and describe model runs. Results focus on model testing and fit, as well as salt content, concentration, and lake level changes with causeway bridge alternatives. We finish by detailing limitations of our approach and discussing major findings and management implications for GSL.

## Background

**Fig 1:**
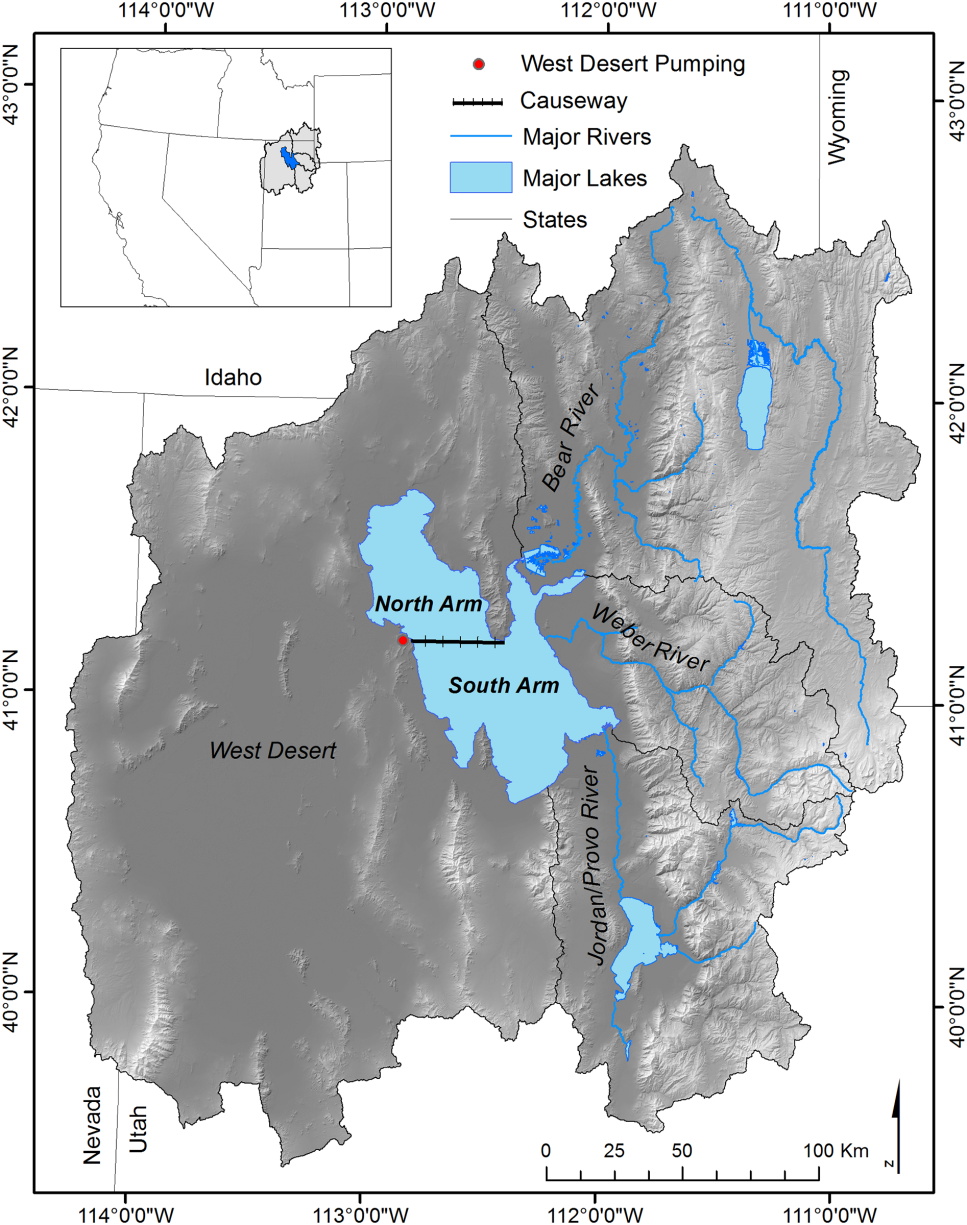
Great Salt Lake and surrounding watershed. Three main tributaries are the Bear, Weber, and Jordan Rivers, which contribute 95% of incoming flow.

GSL is located in north-central Utah and is bounded by the Wasatch and Uinta Ranges to the east and West Desert to the west (Fig 1). The climate is semi-arid. Salt Lake City averages approximately 40 centimeters (cm) of precipitation per year, with the majority of precipitation falling as snow in the Wasatch and Uinta Mountains. Snowmelt-dominated runoff occurs in the spring followed by low flows the rest of the year. The large spring inflow of freshwater is evident in GSL salinity, which is diluted in spring and concentrated in fall and winter [15].

As a terminal lake, GSL’s only outflow of water is via evaporation. GSL surface elevation (henceforth level) is sensitive to inflows and evaporation, and fluctuates through time [5]. Streamflow from the three main tributaries, the Bear, Weber and Jordan Rivers, on average account for approximately 66% of the total freshwater entering the lake, direct precipitation accounts for 31%, and groundwater accounts for the final 3% of inflows [2]. Over the past 160 years, lake level has averaged 1280 m above sea level, and lake area increases dramatically with level. At 1280 m, GSL surface area is 4400 km^2^, however with an increase of elevation to 1283 m, area increases to nearly 6000 km^2^ (using the National Geodetic Vertical Datum of 1929) [1]. Despite its area, the average depth of the lake is only 4.3 m at its mean elevation. The south arm, averaging 1.23 x 10^10^ m^3^ since 1966, is roughly 80% larger than the north arm, which averages approximately 6.75 x 10^9^ m^3^.

Lake level and salinity are inversely related and vary seasonally and decadally with climate. During wet periods, lake level and volume increase, and salinity decreases. During dry periods, lake level and volume decrease, which concentrates salinity. Total minerals, or salts, is the sum of the dissolved and precipitated salts present in the lake and is generally static. Precipitated salt is confined to the north arm, and occurs mostly in dry years. The estimated annual tributary contribution of total dissolved solids (TDS) to GSL is 3.5 million metric tons per year, which is roughly 0.08% of the current 4.5 billion tons of salt in GSL [16]. Thus, in human timescales, tributary salt contributions to GSL are relatively minor.

The total amount of salt in GSL has been reduced over the past half century from mineral extraction and export of lake water from the West Desert Pumping Facility. GSL is an ideal location for mineral extraction via evaporation ponds because of the lake’s high salinity and the region’s dry climate. Four large mineral extraction companies and several smaller companies operate at GSL. Additionally, the West Desert Pumping Facility consists of two large hydraulic pumps that transport brine from the north arm into the adjacent West Desert in wet years to protect local highways and other infrastructure from flooding. The West Desert Pumping Facility operated in wet years 1987 to 1989 and reduced salts by an estimated 0.45 billion metric tons [9]. In total, GSL has lost approximately 1 billion metric tons (~22%) of salt from anthropogenic causes over the past century.

Because the south arm receives most streamflow, the south arm’s lake level has averaged roughly 0.5 m higher than the north arm since the causeway was built, resulting in a pressure gradient which forces brine near the lake surface to flow from the south arm to the north arm. However, since the north is considerably more saline, a density gradient exists at depth within the lake, forcing brine to flow from the north arm to south arm through culverts and causeway fill material [9]. Brine forms a concentrated layer (monimolimnion) below a depth of approximately 6 m in the south arm.

The ecology of GSL’s north and south arms are quite distinct due to salinity differences. The relatively moderate salinity of the south arm supports large populations of brine shrimp (*Artemia franciscana*) and brine fly (*Ephydra cinera*). The hypersaline north arm is largely inhospitable for significant populations of macroinvertebrates, such as *Artemia* or *Ephydra*, to survive. It is instead characterized by several species of phytoplankton and archaea [17]. Although not the only macroinvertebrate present, *Artemia* are a keystone species because they control phytoplankton by grazing, and are also a major food source for birds [18]. However, during wet years with low salinities, predators such as corixids (water boatmen) are able to colonize the south arm. This can result in a trophic cascade where *Artemia* populations fall precipitously, resulting in reduced prey availability for migratory birds and waterfowl, as well as revenue loss for the brine shrimp harvest industry [19]. This occurred in the mid-1980s when salinity levels dropped to nearly 50 g/L in the south arm (compared with average values of 142 g/L). *Ephydra* are another important invertebrate prey item for birds [20], the larvae of which grow on stromatolites (biostromes) in the shallow areas of the south arm [21].

Research into relationships between salinity and production of *Artemia* and *Ephydra* is ongoing, but maximum survival and growth for both species is thought to decrease above 125 g/L [22]. GSL *Artemia* survive with salinity as low as 25 g/L in laboratory experiments [22]; however, as previously noted, predation occurs at higher salinities in GSL. Although salinity is not an exclusive control on *Artemia* or *Ephydra* in GSL, it is a main driver of ecosystem productivity. Thus, we focus on salinity changes from causeway alteration and management in our modeling and analysis. Changes to salinity from causeway alterations are also of keen concern to the brine shrimp harvesting industry, wildlife managers, and mineral extraction companies.

## Methods

### Great Salt Lake Fortran Model

To evaluate the effects of proposed causeway changes on lake elevation, total salt, and salinity, we used USGS’ Great Salt Lake Fortran Model [7–9]. We updated model code to improve flexibility of causeway opening geometry, evaluate longer time series, represent bathymetry to reflect recent evaporation pond development, and estimate salt loss and return from pumping and mineral pond extractions. See White et al. [23] for a thorough description of model code changes.

The model uses a mass balance approach to calculate water and salt flow between GSL’s arms and estimates water volume, total salt, and salinity for each arm of the lake. The GSL Fortran Model assumes water is perfectly mixed within each arm, and does not represent the concentrated deep brine layer. Water volume was calculated at each time timestep (every two days) by:

$$V_{aT}=\;V_{aT-1}+\;QS_{in}+QG_{in}+QC_{in}+\;Q_{wdr}+P-E-QC_{out}-\;Q_{wd}$$ (1)

where V_aT-1_ is water volume of an arm at the previous timestep, QS_in_ is streamflow into the arm, QG_in_ is groundwater inflow, QC_in_ is total flow into the arm through the causeway, P is direct precipitation, Q_wdr_ is return flow from West Desert (if occurring), E is evaporation, Q_wd_ is losses to West Desert pumping (if occurring), and QC_out_ is outflow from an arm through the causeway. Rate variables have units of m^3^d^-1^ and volume variables have units of m^3^.

Mineral content for each arm and timestep was calculated by:

$$L_{aT}=\;L_{aT-1}+L_{T}+L_{inC}+\;L_{rd}-L_{pp}-\;L_{outC}-L_{outP}-\;L_{outE}$$ (2)

where L_aT-1_ is the previous timestep’s salt content, L_T_ is incoming tributary content, L_inC_ is incoming salt content through the causeway, L_rd_ is redissolved content, L_pp_ is precipitated content, L_outC_ is salt content exported through the causeway, L_outP_ is salt content removed when West Desert pumping is initiated, and L_outE_ is content extracted from mineral extractions. Salt content above 350 g/L is converted to precipitated salt, which ignores water temperature effects on salt precipitation. Flows through the culverts and breach are calculated using equations developed by Holley and Waddell [24], Wold et al. [8], and Loving et al. [9]. Details of equations are summarized in Loving et al. [9]. All salt losses/additions are in metric tons, and salinity is calculated as C_aT_ = L_aT_/V_aT_ in units of g/L.

Holley and Waddell [24] did not anticipate causeway culverts to be submersed, and therefore did not develop equations for bi-directional flow with submerged conditions. Submerged conditions occurred prior to 1997 when Wold et al. [8] updated the model. However since the culverts typically are inundated with debris when submersed, they assumed no flow occurred through culverts when submerged. At those times, bi-directional flow occurs only through the breach and fill material. Bi-directional flow through porous causeway fill material occurs over its length (approximately 35 km), while the two culverts combined are 0.03 km. Thus, when culverts are submerged and plugged with debris, their influence on bi-directional flow is likely minimal. Loving et al. [9] updated the original equations developed by Holley and Waddell [24] to calculate bi-directional flow with submerged culverts. However, due to the tendency for culverts to become plugged with debris when submerged, flow measurements to verify the new equations were not taken when culverts were submerged. Both Wold et al. [8] and Loving et al. [9] agreed that flow through the culverts during this time was greatly diminished. Despite the equations developed Loving et al. [9] for bi-directional flow with submerged culvert conditions, we found the model to be considerably more accurate by assuming no culvert flow when culverts are submerged, though in reality, some amount of bi-directional flow surely occurs when the culverts are submerged. Unsubmerged culvert flow equations have been previously described in Holley and Waddell [23] and submerged culvert flow equations in Loving et al. [8].

### Input Data and Sources

Daily streamflow contributions were obtained from USGS gages on the three major rivers feeding the lake, the Bear, Jordan, and Weber Rivers [5]. The Bear River Bay is hydrologically connected to the south arm through an opening in the Bear River Bay Bridge. The minor ephemeral streams that contribute approximately 5–7% of streamflow were ignored here. Direct precipitation was from Oregon State University’s PRISM dataset [25], using a 2.5 arc min (~ 4 km) grid. Precipitation varied between the north and south arms by averaging grid cells that fell within the north or south arm of GSL [5]. Groundwater was assumed constant at 10 million m^3^/ month in the south bay and 1 million m^3^/month in the north bay [9]. Monthly evaporation was estimated by closing a mass balance equation with changing volume and inflows. Mohammed and Tarboton [5] previously completed an extensive analysis comparing GSL evaporation estimates using a mass balance approach or salinity-adjusted Penman equation [26]. They found the mass balance approach more accurately reproduced historical lake level, salt content, and salinity, compared to using a salinity-adjusted Penman equation [5,26].

Causeway opening geometry including the culverts, breach, and proposed bridge designs were from Union Pacific Railroad [6], and causeway subsidence rates were from Loving et al. [9].

### Model Runs

Seven model runs simulating 1966–2012 were conducted, using identical climate, streamflow, West Desert Pumping, mineral extraction, initial lake elevation, and total salt data. The 46 years of historical data represent historical climate variability to estimate effects of causeway modifications on GSL. Historical data are stationary and do not represent anticipated climate warming, nor do they represent changing water withdrawals from population growth and urban development. Details of each model run are summarized in Table 1 and described below.

**Table 1:** Model runs with causeway and bridge design details (if applicable).

Model Name	Number of culverts	Breach	Subsidence	New bottom Width (m)	New Bottom Elevation (m)
Historical	2	Opened in 1984	Subsides over time	NA	NA
Alternative A	0	Opened in 1984	Subsides over time	18.6	1273.5
Alternative B	0	Opened in 1984	Subsides over time	9.4	1273.5
Alternative C	0	Opened in 1984	Subsides over time	14.9	1275
Alternative D	0	Opened in 1984	Subsides over time	20.1	1276.5
Current Conditions	0	Open throughout	Fully subsided	NA	NA
Whole Lake	0	No breach	No causeway	NA	NA

#### 1. Historical conditions

The historical 1966–2012 run simulates salt and water balance with the following causeway changes occurring through time. The causeway and culverts subsided, and flow through causeway material was reduced in the late 1970s following subsidence [9]. The breach was deepened in 1998 and again in 2000, by 4.2 m and 2.1 m, respectively. Salt losses occurred from mineral extraction and 1987–1989 West Desert Pumping. This run simulates historical conditions to evaluate model fit and accuracy, and provides a reference comparison for other model runs.

#### 2–5. Union Pacific Railroad bridge alternatives

**Fig 2:**
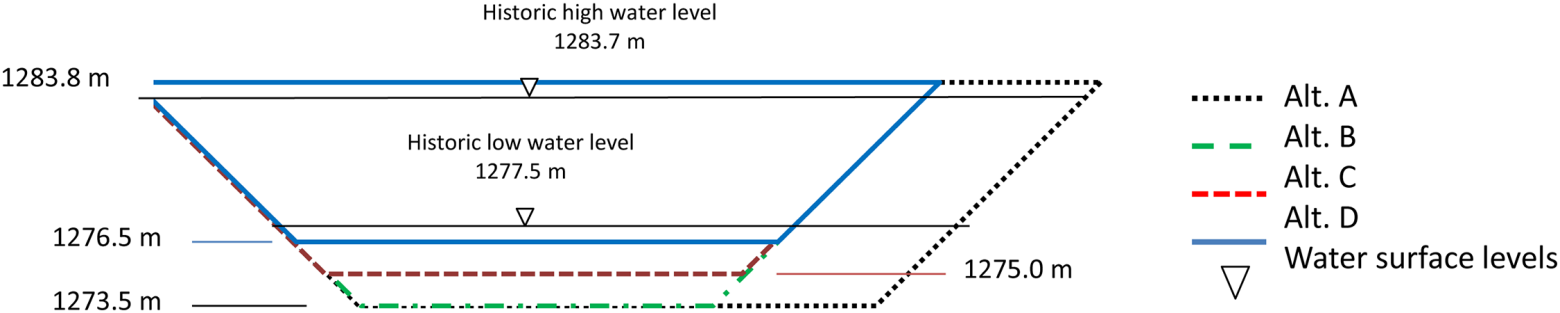
Bridge alternative designs [6].

These runs estimate water and salt flow through the causeway if a bridge is built to replace closed culverts. Union Pacific Railroad proposed four trapezoidal bridge alternatives (Table 1 and Fig 2). Alternative A is the largest. Alternatives B, C, and D, are 10 m narrower than alternative A with identical top widths and elevations, but alternative B has the same bottom depth as alternative A, while alternatives C and D are 1.5 m and 3 m shallower, respectively [6]. The location of the bridge opening in the causeway does not change between alternatives. All bridge alternatives use identical equations (with different parameters based on size and design) to calculate bi-directional flow through the bridge opening. Head and density differentials calculate flow in a trapezoidal opening. The same equations are used to calculate flow through the breach [9].

#### 6. Current conditions

The current conditions run simulates causeway conditions when culverts are closed, the causeway has subsided, and flow through causeway material is reduced. This run estimates lake level and salinity if a bridge is not built to replace closed causeway culverts, representing lake conditions subsequent to December 2013, after both culverts were filled.

#### 7. Whole lake conditions

A whole lake with no causeway was estimated by dividing the sum of north, south, and precipitated salt by the combined volume of each arm. These calculations were completed with the statistical program R [27] using data from the historical model run. Salt losses from pumping and mineral extractions are included in the whole lake condition so this run is comparable to other alternatives.

## Results

### Model Calibration

**Fig 3:**
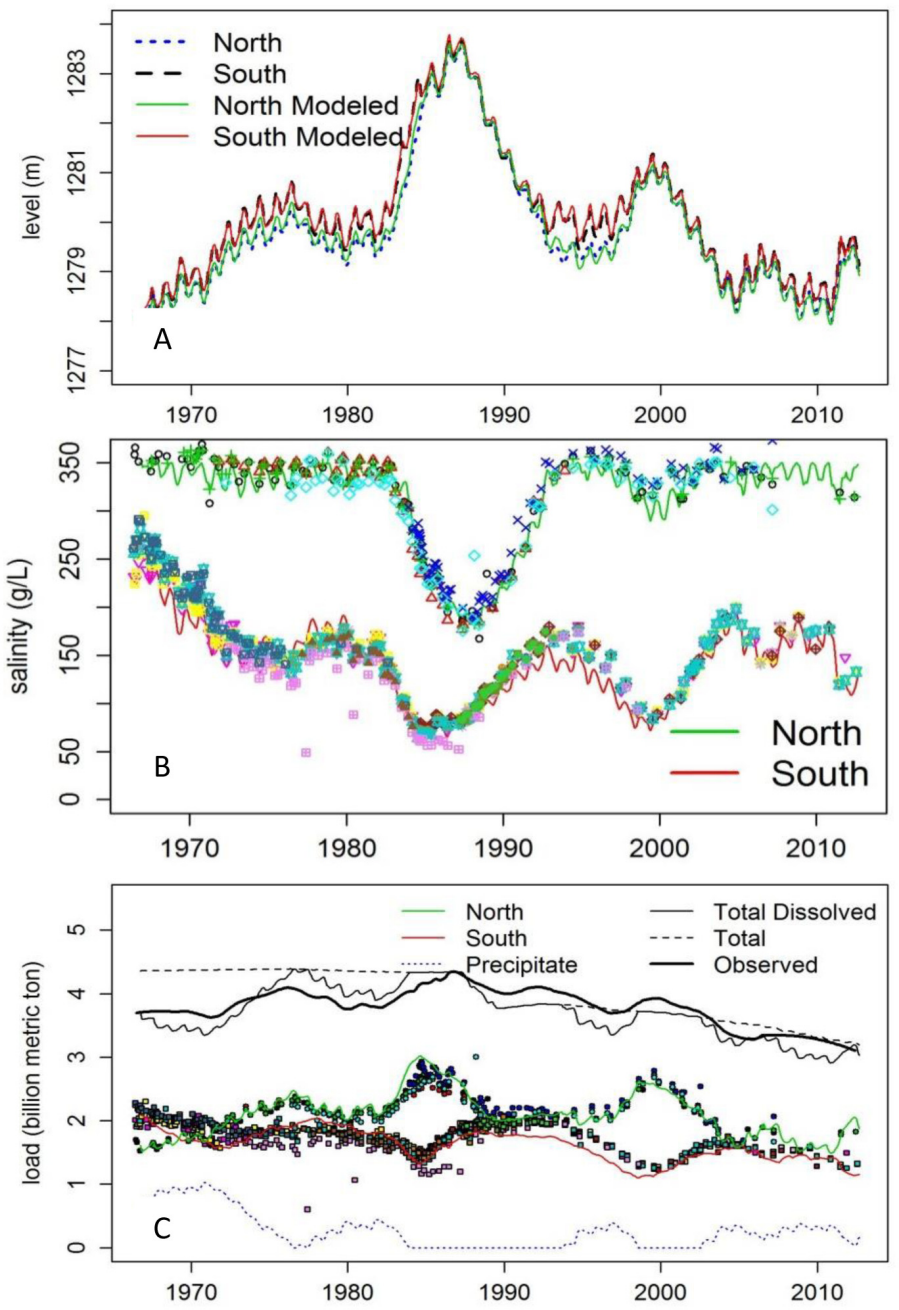
Measured and modeled historical A) lake level, B) total salt, and C) salinity in GSL north and south arms. Points show measured USGS data at various locations.

Overall, our model provides an excellent representation of GSL lake level, salt content, and salinity (Fig 3). Modeled and measured data track well and there is no consistent bias. However, from 1989–2000 both modeled salt content and salinity in the south arm are lower than observed data. During that time, culverts were submerged and we assumed no water flowed through them. In reality, the elevation and the density gradients between the north and south arms would likely have exchanged some small and unmeasured quantity of water in both directions through the culverts.

The Nash-Sutcliffe Efficiency (NSE) statistic evaluates the predictive power of models by comparing the magnitude of modeled residual variance with measured variance [28–30]. This unitless statistic ranges from -∞ (no fit) to 1 (perfect fit). Average annual lake level is modeled with near complete accuracy (0.99 for both the north and south arms). Average annual salinity in each arm is also excellent with values of 0.94 and 0.89 in the north and south arm, respectively. Average annual total salt content is less accurate, with NSE of 0.78 in the north, and 0.36 in the south. The intent of NSE is to quantify the model's ability to explain variability. The total amount of salt in GSL is, for all intents and purposes, constant. This, in part, explains the high NSE values for lake level and poor NSE for the total amount of salt. Salt movement between the arms gives rise to the small variability in total salt in each arm, and the small observed variability that appears in the denominator of NSE leads to poorer values. This effect can be observed in Fig 3C, where the amount of salt in each arm is generally flat and the difference between modeled and observed is of comparable scale to the observed variability. On the other hand, lake level is significantly variable and the model tracks this well, leading to high NSE values.

Periods when culverts were submerged (and we assumed no bi-directional flow) coincide with the least accurate model fit. As noted, some flow likely occurred during this period, but assuming zero flow was more accurate for lake level and salinity than utilizing previous equations derived for submerged conditions. Additional uncertainty exists regarding salt loss estimation from West Desert Pumping. The period that the model is least accurate begins around 1990, immediately following pumping activity. Finally, total salt content is not a direct measurement, rather a calculation based on ionic concentration and lake volume; therefore it has the highest variability and least certainty of all modeled variables.

There is a reduction in total salt content of roughly 1 billion metric tons from 1985–2012 (Fig 3C) in our model. Roughly half (0.45 billion metric tons) of this loss occurred in the late 1980s, when brine was pumped to the West Desert to evaporate. The remaining losses are from commercial mineral extractions. The net loss of salt manifests in salinity levels in the north arm when it is unsaturated so no precipitated salt is present.

### Bridge Alternatives

**Fig 4:**
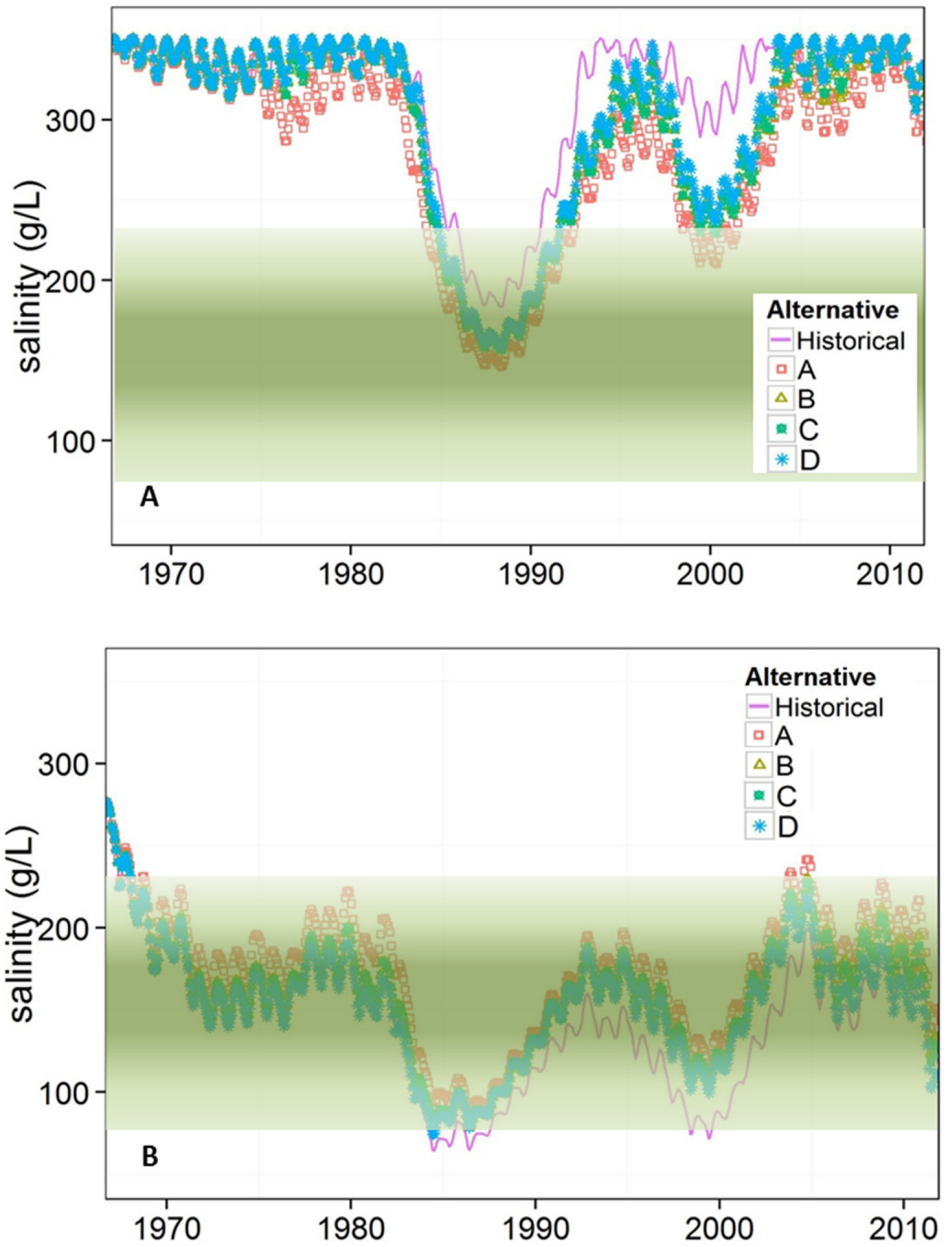
Modeled salinity concentrations of simulated historical conditions and bridge alternatives in GSL A) north arm and B) south arm. Green bands show approximate brine shrimp salinity thresholds, although uncertainty exists regarding exact concentrations that negatively affect brine shrimp [21].

**Fig 5:**
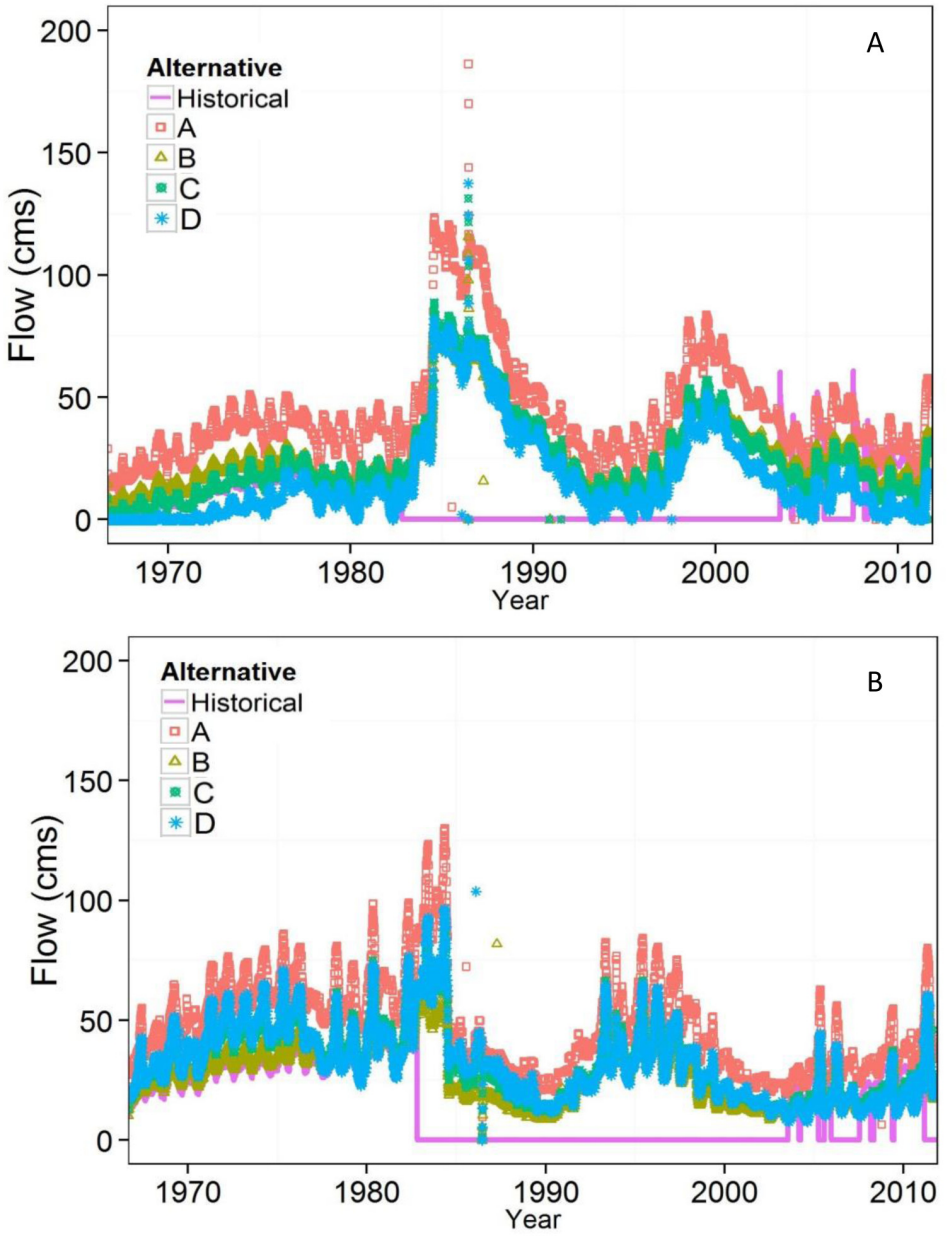
Bi-directional flow from A) north to south, and B) south to north with bridge alternatives and historical conditions. Historical culvert flow is zero when culverts were submerged (1984–2004). Bridge alternatives were never submerged.

Differences in salinity between the proposed bridge designs and historical conditions were greatest from the mid-1980s through mid-2000s (Fig 4), which coincided with the time that culverts were submerged (Fig 5). Alternative A, the largest bridge design, allowed for the greatest bi-directional flow exchange (Fig 5) while alternative D allowed the least. Alternatives B and C were nearly identical throughout the modeled period. The top elevation (1284 m) of all bridge alternatives was sufficiently high so that they were never submerged with 1966–2012 historical hydrology. Summary salinity statistics for each model run are shown in Table 2.

**Table 2:** Mean, maximum, and minimum salinity in the north and south arms for all model runs.

Model Run	North Arm			South Arm		
	Mean salinity (g/L)	Max salinity (g/L)	Min salinity (g/L)	Mean salinity (g/L)	Max salinity (g/L)	Min Salinity (g/L)
Historical	317	351	183	142	276	64
Current Condition	320	351	190	125	276	44
Alternative A	282	351	146	173	277	86
Alternative B	297	351	159	160	276	79
Alternative C	297	351	156	159	276	80
Alternative D	301	351	159	152	276	74
Whole Lake	222 (mean)		115 (min)		351 (max)	

**Fig 6:**
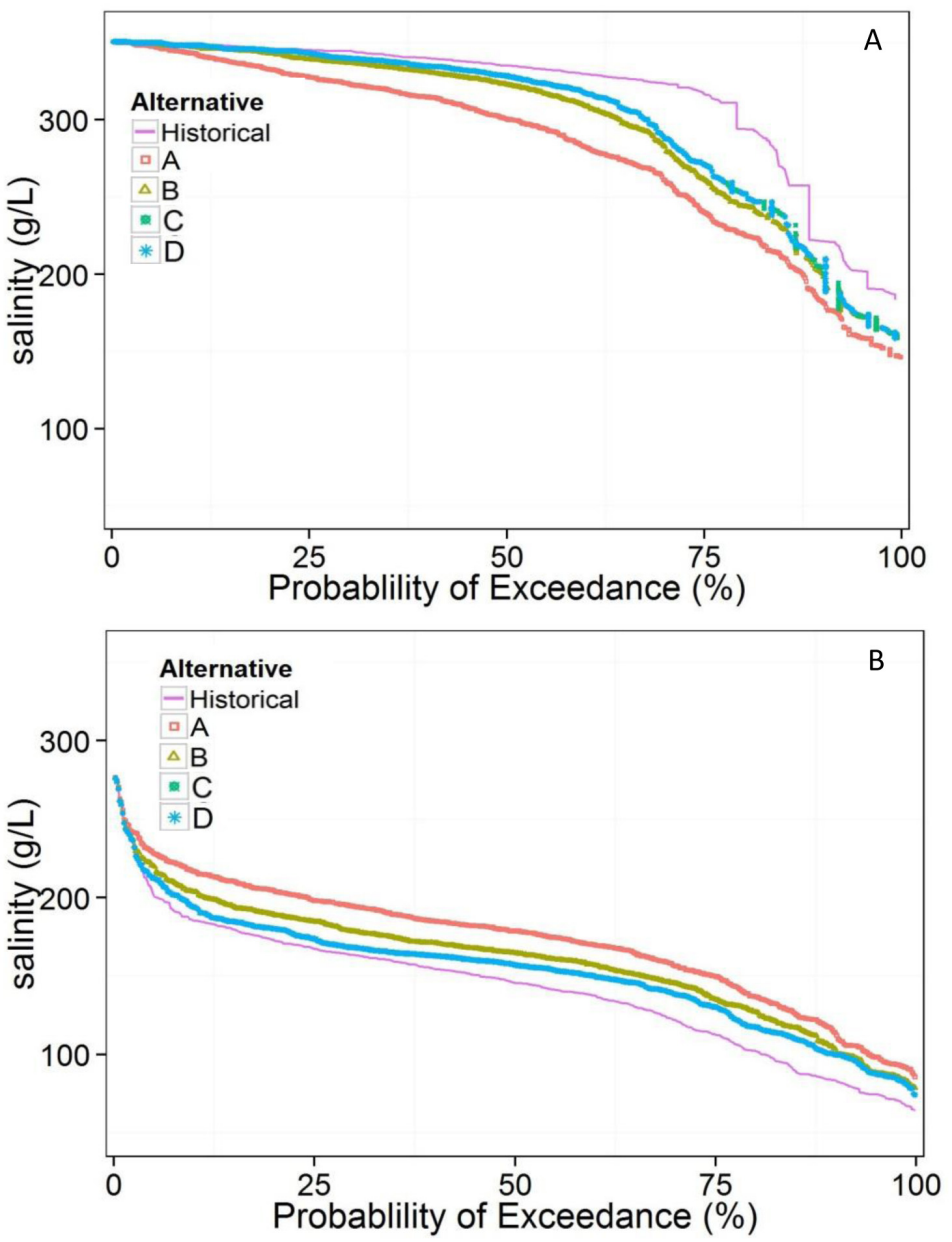
Exceedance probabilities of historical conditions and bridge alternatives for GSL A) north arm and B) south arm.

Probability exceedance curves from the modeled period indicate that any bridge opening in the causeway increases salinity in the south arm and reduces it in the north arm (Fig 6). The 50^th^ percentile salinity in the south arm increased from 150 g/L historically, to 180 g/L with bridge alternative A, and 167 g/L, 165 g/L, and 157 g/L for bridge alternatives B, C, and D, respectively. Similarly, salinity decreased in the north arm, where the 50^th^ percentile drops from 335 g/L historically to 290 g/L, 315 g/L, 316 g/L, and 321 g/L for bridge alternatives A, B, C, and D respectively.

**Fig 7:**
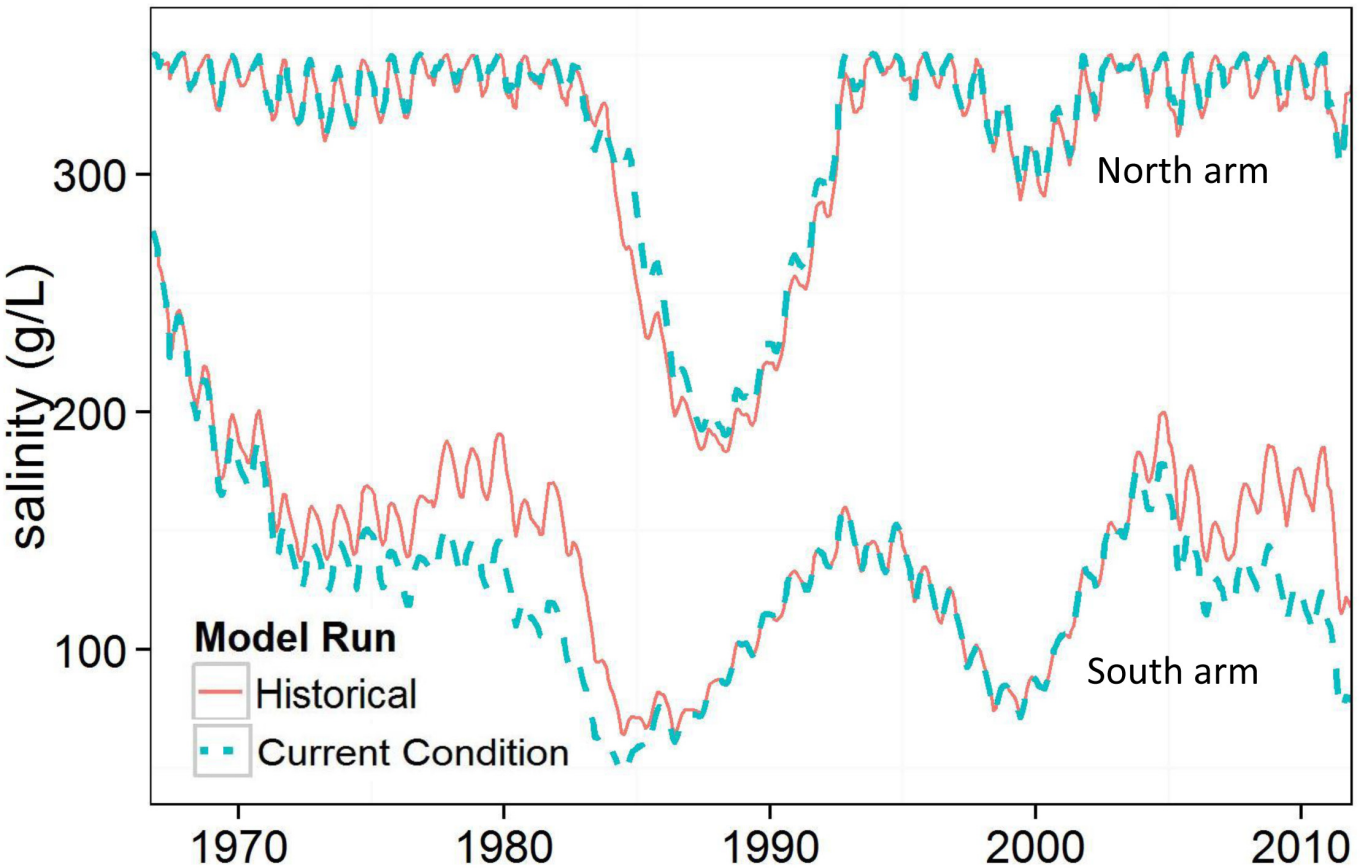
Historical and current conditions salinities for GSL north and south arms. Current conditions has closed culverts and a subsided causeway.

Among the four proposed bridge designs, alternative A, with the largest opening, is the most distinct and most resembles estimated whole lake (no causeway) conditions (Fig 7). This suggests that the width of causeway opening near the lakebed is important for increasing bi-directional flow between the arms. Although bridge alternative B is nearly 1.5 m shallower than alternative C, both result in nearly identical salinities in the north and south arms throughout the modeled period (Fig 5). Alternative D, with the shallowest bridge bottom, has salinities most similar to those of the historical model run. An exception occurs from 1984–2004, when the culverts in the historical conditions run were inundated. Despite close alignment of flow and salinity throughout much of the period, there is a systematic shift to more moderate salinities in each arm during this period with alternative D compared to historical conditions.

### Current Conditions

The current conditions model run simulated lake conditions with closed culverts and a subsided causeway throughout the 46-year modeled period. Overall, the salinity gradient between GSL’s north and south arms became more pronounced with current causeway conditions (Fig 7). Salinity sometimes decreased in the south arm with no noticeable increase in the north arm because salt concentrations above 350 g/L are converted to precipitated salts.

When the current conditions run is compared to historical conditions, which had two culverts and slowly subsiding causeway reducing permeability of fill material in the late 1970s, salinity diverged between the two runs most when culverts were not submerged in the historical run (1966–1984 and 2005–2012). Bi-directional flow through the culverts contributed to water mixing between arms in the historical conditions model run. Differences in south arm salinity between model runs varies through time. A large salinity difference occurred from 1973–1984 when bi-directional flow occurred through the culverts in the historical run, but flow through causeway fill provided the only flow exchange in the current conditions simulation (Fig 7). A similar divergence occurred in 2005–2012. When the breach was installed in 1984 in each model, salinity converged somewhat, highlighting the utility of causeway openings to equalize salinity.

### Whole Lake

**Fig 8:**
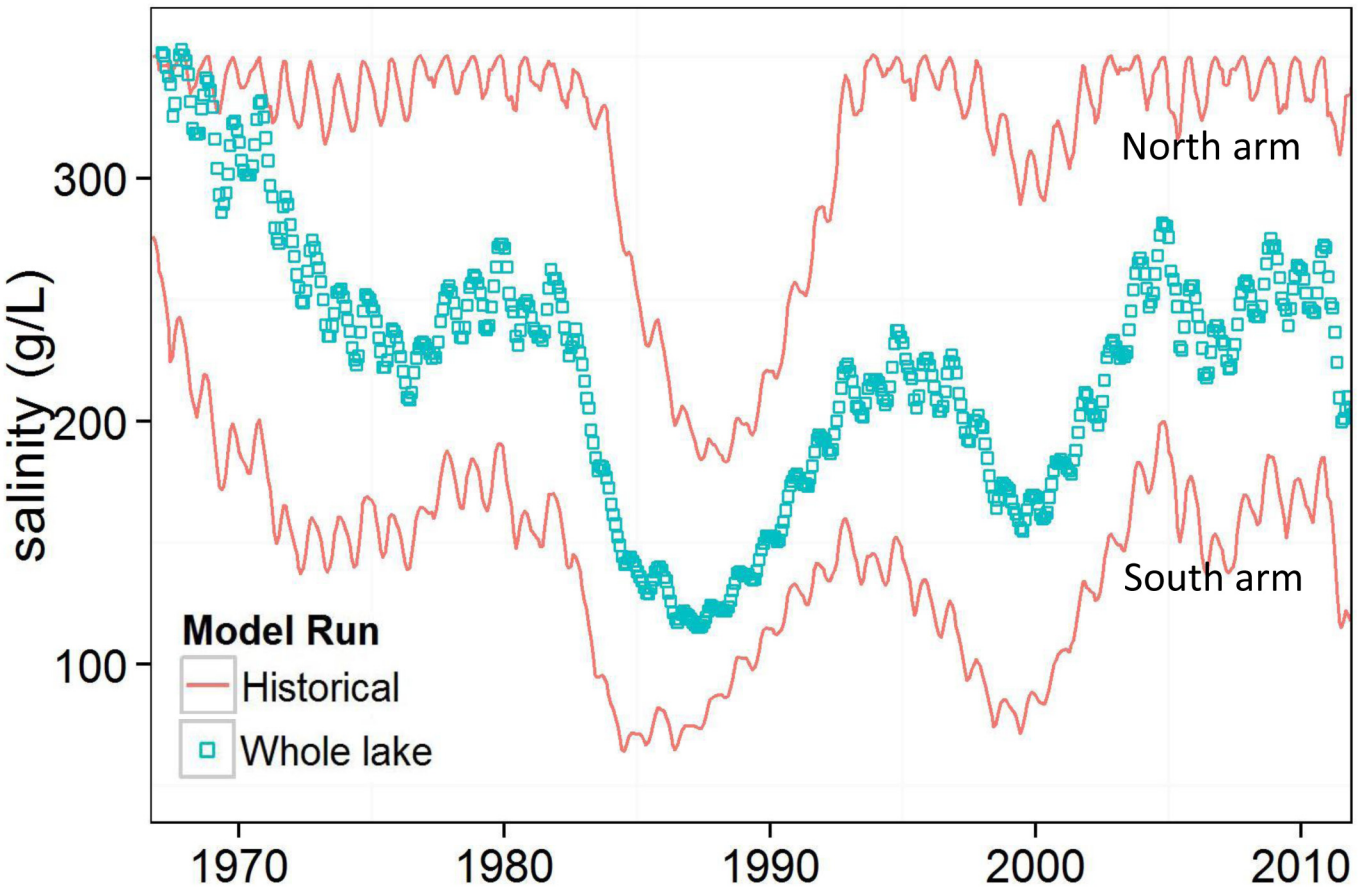
Historical north and south arms, and whole lake salinities.

Estimated whole-lake salinity, which assumes a causeway was never built, is shown in Fig 8. Estimated whole lake salinity was more moderate than historical conditions, typically remaining between approximately 115 g/L to 225 g/L. The south arm is roughly twice the volume as the north arm, so whole lake salinity trends toward historical south arm salinities. These results are consistent with previous estimates of whole lake GSL conditions [9,31]. Although our model assumed well-mixed conditions, in reality a GSL without a causeway would have some variability vertically in the water column and would also be fresher near stream confluences.

### Limitations

This study focuses solely on changes to salinity and water balance from modifications to the causeway bisecting GSL. Although these changes are likely to have significant effects on the economic and ecological uses of GSL, quantifying those effects are beyond the scope of this study.

Our modeling assumes historical hydroclimate conditions for precipitation, evaporation, and streamflow. We do not consider climate change, although climate-induced alterations to hydrology are expected over the coming century [32]. Future climate is unlikely to mimic historical conditions, and further research is needed to assess how causeway modifications will affect GSL level and salinity with anticipated climate change. Also, population growth and ongoing water development along the Wasatch Front may increase water demands by depleting streamflow contributions to GSL. This is another important topic that may significantly alter GSL hydrology, ecology, aesthetics, and economic benefit, and merits additional research.

Some model runs were affected by the wet years of the mid-1980s, when high lake levels caused culverts to be submerged and bi-directional culvert flow was assumed to be zero. As discussed above, historical inter-arm flow during this period was greatly reduced, but likely not zero. Flow through causeway fill is considered to be uniform along the length of the causeway. Wold et al. [8] found that bi-directional flows are lowest at each end of the causeway and slowly increase towards the middle. Despite this spatial variability, Wold et al. [8] and Loving et al. [9] used homogenous causeway permeability with sufficient accuracy to replicate lake behavior. These are limitations of this model and potential improvements to make for future modeling studies.

The model assumes that each arm is perfectly mixed. In reality, spatial variability exists within each arm. The most obvious and important example of this is the monimolimnion, or vertically-stratified deep brine layer in the south arm, which exhibits salinity close to that of the north arm. This layer is dense although it is believed that some mixing occurs between it and the fresher water resting atop it [33,34]. Variable salinities within the south arm also exist at bays where tributaries flow into GSL. Although spatial variability occurs, measured salinities at various locations of each arm support the assumption that water is well mixed in the shallow brine layer (Fig 3). Overall, our modeling and analysis provides direct and useful comparisons of alternative causeway modifications and designs.

## Discussion and Management Implications

With construction of a solid-fill railroad causeway in 1959, GSL hydrology and salinity were dramatically changed, affecting the ecological, social, and economic uses of GSL. Lake managers and stakeholders are keenly interested in the future condition of GSL following culvert closures in 2012 and 2013. Our modified USGS GSL Fortran Model simulates GSL water and salt balance with proposed causeway bridge openings to estimate future lake conditions.

GSL’s current conditions, with closed culverts and reduced interflow through a subsided causeway, will increase salinity differences between the north and south arms. Average predicted salinities for the south arm and north arm with current conditions are 125 g/L and 320 g/L, respectively—a decrease of roughly 11% in the south and an increase of 1% in the north from historical causeway conditions. The south arm will become increasingly fresh from streamflow contributions, and the north arm will become increasingly saline with precipitation as the primary inflow of freshwater. Thus, current conditions for GSL will likely lead to poor macroinvertebrate habitat in the north arm and a potential reduction of macroinvertebrate habitat in the south arm.

The four proposed bridge designs create different salinity conditions in the north and south arms. If replication of culvert flow is the primary objective, alternative D is best (Fig 4). In fact, alternative D will improve upon the culvert design because the top elevation is higher so it is less vulnerable to lake inundation. However, the culverts were designed for boat passage between the bays, without considering specific flow or salinity conditions [23]. Therefore, replicating flow through culverts may not result in preferred conditions for lake ecology, mineral extraction industries, or brine shrimp harvesters. If maximizing inter-arm flow exchange is the goal, alternative A is best (Fig 5). With this alternative, average salinity is reduced by 35 g/L in the north arm and increased by 31 g/L in the south arm, compared to historical conditions. Alternative A is the most similar to average whole lake salinity of 222 g/L, which estimates natural conditions without the railroad causeway.

Like most terminal lakes, GSL has multiple and competing uses. Even within user groups, causeway modification may have non-uniform consequences. Commercial mineral extraction, for example, occurs in the north and south arms. Thus, those operating in the south would welcome salinity increases provided by larger bridge designs, while those in the north would lament the loss of minerals available for extraction if salinity was less than saturation. Similarly, increasing south arm salinity through a larger bridge opening would favor brine shrimp survival (and brine shrimp harvest) when lake levels are high and salinity drops enabling freshwater predator invasion. However, larger bridge openings could also result in the south arm being too salty for brine shrimp in some years. GSL management decisions will be difficult and modeling analyses such as this one help to simplify decision-making.

A bridge opening design that is adaptive to changing future conditions or objectives would be useful. Adaptive management strategies, such as controllable gates or adjustable depths between bays, have been informally discussed amongst interests groups on GSL [31]. These options allow for salinity control depending on conditions and needs. However, modeling such systems was outside the scope of this study.

Our results show that causeways, or other hydrologic separations in terminal lakes, can significantly change salt balance. The magnitude of these changes can be partially controlled with causeway management. Such a strategy may be useful for other terminal lakes worldwide, particularly those facing desiccation and increasing salinity. Strategic isolation of parts of terminal lakes may provide an opportunity to maintain lower (and presumably preferred) salinity levels in some portions of the lake. Using a relatively simple mass balance model, such as the one described here, provides a method to evaluate such opportunities.

Determining how to manage terminal lake elevation and salinity are emerging branches of ecological management and water resources management [1]. Many terminal lakes are threatened worldwide. Some terminal lakes have similar causeways, such as Iran’s Lake Urmia and central Asia’s Aral Sea, others have inflow and salinity alterations from upstream water diversions, such as California’s Mono Lake, Nevada’s Walker Lake, and Central Asia’s Aral Sea. We contribute to that knowledge by quantitatively assessing water and salt flow for a specific terminal lake, Utah’s GSL. This research illustrates that flow alterations and flow modifications within terminal lakes cannot be separated from lake salinity, ecology, management, and economics.
